# Towards Complete Tumor Resection: Novel Dual-Modality Probes for Improved Image-Guided Surgery of GRPR-Expressing Prostate Cancer

**DOI:** 10.3390/pharmaceutics14010195

**Published:** 2022-01-14

**Authors:** Maryana Handula, Marjolein Verhoeven, Kuo-Ting Chen, Joost Haeck, Marion de Jong, Simone U. Dalm, Yann Seimbille

**Affiliations:** 1 Department of Radiology and Nuclear Medicine, Erasmus MC, University Medical Center Rotterdam, 3015 GD Rotterdam, The Netherlands; m.handula@erasmusmc.nl (M.H.); m.verhoeven.1@erasmusmc.nl (M.V.); s.dalm@erasmusmc.nl (S.U.D.); 2 Department of Chemistry, National Dong Hwa University, Shoufeng, Hualien 974301, Taiwan; ktchen26@gms.ndhu.edu.tw; 3 AMIE Core Facility, Erasmus MC, University Medical Center Rotterdam, 3015 GD Rotterdam, The Netherlands; j.haeck@erasmusmc.nl; 4 Life Sciences Division, TRIUMF, Vancouver, BC V6T 2A3, Canada

**Keywords:** prostate cancer, PC-3, GRPR, NeoB, dual-modality imaging, IEDDA, sCy5, pre- and intraoperative imaging

## Abstract

Nuclear and optical dual-modality probes can be of great assistance in prostate cancer localization, providing the means for both preoperative nuclear imaging and intraoperative surgical guidance. We developed a series of probes based on the backbone of the established GRPR-targeting radiotracer NeoB. The inverse electron demand of the Diels–Alder reaction was used to integrate the sulfo-cyanine 5 dye. Indium-111 radiolabeling, stability studies and a competition binding assay were carried out. Pilot biodistribution and imaging studies were performed in PC-3 tumor-bearing mice, using the best two dual-labeled probes. The dual-modality probes were radiolabeled with a high yield (>92%), were proven to be hydrophilic and demonstrated high stability in mouse serum (>94% intact labeled ligand at 4 h). The binding affinity for the GRPR was in the nanomolar range (21.9–118.7 nM). SPECT/CT images at 2 h p.i. clearly visualized the tumor xenograft and biodistribution studies, after scanning confirmed the high tumor uptake (8.47 ± 0.46%ID/g and 6.90 ± 0.81%ID/g for probe [^111^In]In-**12** and [^111^In]In-**15**, respectively). Receptor specificity was illustrated with blocking studies, and co-localization of the radioactive and fluorescent signal was verified by ex vivo fluorescent imaging. Although optimal tumor-to-blood and tumor-to-kidney ratios might not yet have been reached due to the prolonged blood circulation, our probes are promising candidates for the preoperative and intraoperative visualization of GRPR-positive prostate cancer.

## 1. Introduction

Worldwide, prostate cancer (PCa) is the second most frequently diagnosed cancer among men, with about 1.4 million new cases in 2020 alone [1]. The surgical removal of the prostate gland, in whole or in part, combined with pelvic lymph node dissection is one of the most widely used treatment options to cure localized PCa [2]. Although successful in many cases, the recurrence rate after radical prostatectomy is still as high as 20–40% [3]. One of the indicators for an increased risk of relapse is the observation of positive surgical margins [4]. As well as the multifocal nature of many primary prostate tumors, the need to maintain physiological functions, such as potency and continence, constitutes an additional challenge for surgeons [5,6]. Such a nerve-sparing surgical approach is a complex procedure and may therefore come at the expense of complete tumor eradication [7].

To reduce recurrence and, therefore, the need for secondary treatment, new imaging techniques that provide accurate surgical guidance may offer a solution. Over the years, fluorescence-guided surgery has emerged as a powerful tool for real-time intraoperative imaging in the surgical field [8]. To enable the precise removal of cancer tissue, receptor-targeting agents coupled to a fluorescent dye can lead to improved tumor localization [8]. Fluorescent tumor-targeting tracers have a high spatial resolution but are limited by their low tissue penetration. This is where nuclear medicine can play a pivotal role, as a radioisotope can be used for non-invasive preoperative nuclear imaging that supports surgical planning, as well as for the determination of the approximate localization of deeper lesions intraoperatively, with high sensitivity [9]. The benefits of the complementary use of a radioactive and fluorescent signal for image-guided surgery have led to an increased interest in the development of nuclear and optical dual-modality probes [10,11].

In nuclear medicine, recent advances in PCa-targeting radiotracers have provided a range of promising vectors for tumor targeting. One of the aberrantly overexpressed targets in PCa is the gastrin-releasing peptide receptor (GRPR) [12,13]. Imaging studies with GRPR-targeted radiotracers have demonstrated high tumor uptake and excellent visualization of tumor lesions in cancer patients [14,15,16,17]. NeoB (formerly known as NeoBOMB1) is one such established radiotracer, with a high binding affinity for GRPR, and is favorable for in vivo pharmacokinetics [18,19]. NeoB is therefore an excellent molecule to serve as a basis for the development of a dual-modality probe.

In this study, we integrated the sulfo-cyanine 5 fluorescent (sCy5) dye into the DOTA-coupled GRPR antagonist NeoB, using the inverse electron-demand Diels–Alder reaction (IEDDA) [20,21]. A tetrazine (Tz) moiety was coupled to the fluorescent dye and a *trans*-cyclooctene (TCO) group was incorporated to the backbone of NeoB via an additional lysine residue. A linker (*p*ADA or PEG_4_) was introduced between the binding domain and the DOTA chelator, in combination with or without a PEG_4_ linker between the TCO group and the lysine. The methodological approach taken in this research resulted in a panel of two single- and four dual-modality probes. After the development of these compounds, we assessed the effect of the dye attachment on the stability and affinity for GRPR in vitro and the tumor-targeting capability and biodistribution in vivo. With this study, we aim to demonstrate the potential of the novel GRPR-targeting dual-modality probes for preoperative and intraoperative PCa visualization.

## 2. Materials and Methods

### 2.1. General Information

All chemicals and solvents were obtained from commercial suppliers and used without further purification unless specified. DOTA-tris(*t*Bu)ester was purchased from Macrocyclics (Plano, TX, USA) and ^111^InCl_3_ (370.0 MBq/mL in HCl, pH 1.5–1.9) was provided by Curium (Petten, The Netherlands). Fmoc-based solid-phase peptide synthesis (SPPS) of peptide (**1**) was conducted on a CS136 automated peptide synthesizer (C.S. Bio, Menlo Park, CA, USA). High-performance liquid chromatography (HPLC) was carried out on a Waters 2659 series system (Etten-Leur, The Netherlands) equipped with a diode array detector and a radio-detector made by Canberra (Zelik, Belgium). Low-resolution electrospray ionization (ESI) mass spectra were recorded on a TSQ Quantum UltraTM triple quadrupole mass spectrometer from Thermo Fisher Scientific (Lansingerland, The Netherlands). Nuclear magnetic resonance (NMR) spectra were recorded in D_2_O on a Bruker AVANCE 400 (Leiden, The Netherlands) at an ambient temperature. Chemical shifts are given as *δ* values in ppm, and coupling constants *J* are given in Hz. The splitting patterns are reported as s (singlet), d (doublet), t (triplet), q (quadruplet), qt (quintuplet), m (multiplet), and br (broad signal). Instant thin-layer chromatography (iTLC) plates, on silica gel impregnated glass-fiber sheets, were eluted with sodium citrate (0.1 M, pH 5). The plates were analyzed by a bSCAN radio-chromatography scanner from BrightSpec (Antwerp, Belgium), equipped with a sodium iodide detector. The radioactive samples used for the determination of Log D_7.4_, in vitro assays, and in vivo uptake in tissues were counted using a Gamma counter, Wizard 2480 (Perkin Elmer, Waltham, MA, USA). Activity measurements were performed using the VDC-405 dose calibrator (Comecer, Joure, The Netherlands). The analysis of the products was performed by HPLC on an analytical column (Gemini^®^, Phenomenex C-18, 5 μm, 250.0 × 4.6 mm) with a gradient elution of acetonitrile (ACN) (5% to 95% in H_2_O, containing 0.1% TFA) at a flow rate of 1 mL/min over 30 min. Purification of the peptides was performed using a semi-preparative column (Luna^®^, Phenomenex C-18, 5 μm, 250.0 × 10.0 mm), with a gradient elution of ACN (10% to 90% in H_2_O) at a flow rate of 3 mL/min over 30 min.

### 2.2. Chemistry and Radiochemistry

#### 2.2.1. Synthesis of fQWAVGH (**1**)

fQWAVGH (**1**) was synthesized using an *N*^𝛼^-Fmoc solid-phase peptide synthesis strategy. The conjugation of the Fmoc-protected (**1**) sequence (D-Phe-Gln(Trt)-Trp(Boc)-Ala-Val-Gly-His(Trt)) to the 2-chlorotrityl chloride resin was carried out in dimethylformamide (DMF) with 2-(1*H*-benzotriazol-1-yl)-1,1,3,3-tetramethyluronium hexafluorophosphate (HBTU) (3.9 equiv.), Oxyma Pure (4 equiv.) and *N*,*N*-diisopropylethylamine (DIPEA) (8 equiv.) for 1 h. Fmoc deprotection was accomplished by treatment of the resin with a 20% solution of piperidine in DMF. Amide formation and Fmoc deprotection were monitored using the Kaiser test. Double couplings were performed when the reaction was not complete. The peptide synthesis was initiated by loading Fmoc-His(Trt)-OH (0.5 mmol, 1 equiv.) onto the 2-chlorotrityl chloride resin (2 g, average loading capacity: 1.6 mmol/g). The resin was shaken for 90 min at room temperature (rt). Then, the resin was capped using dichloromethane/methanol/*N*,*N*-diisopropylethylamine (DCM/MeOH/DIPEA) (20 mL, *v*:*v*:*v* = 80:15:5) for 15 min at rt. Subsequent Fmoc deprotection and coupling with Fmoc-Gly-OH (4 equiv.), Fmoc-Val-OH (4 equiv.), Fmoc-Ala-OH (4 equiv.), Fmoc-Trp(Boc)-OH (4 equiv.), Fmoc-Gln(Trt)-OH (4 equiv.) and Fmoc-D-Phe-OH (4 equiv.) were achieved following the same protocol described above.

#### 2.2.2. TCO-pADA-fQWAVGH-NHCH[CH_2_CH(CH_3_)_2_]_2_ (**4a**)

**Scheme 1:**
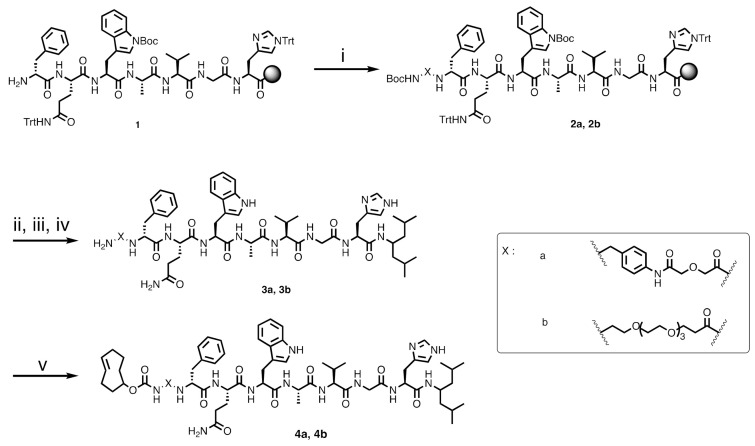
Synthesis of **4a** and **4b**. Reagents and conditions: (**i**) linker a or b, HBTU/Oxyma Pure, DIPEA, 2 h, rt; (**ii**) HFIP/DCM, 1 h, rt; (**iii**) PyBOP, DIPEA, 4-amino-2,6-dimethylheptane, DMF, 1 h, rt; (**iv**) TFA/H_2_O/TIS, 1 h, rt; (**v**) TCO-NHS, Et_3_N, H_2_O/ACN, 1 h, rt.

Once the peptide sequence was completed, the Boc-NH-*p*ADA-OH linker was coupled to the peptide (Scheme 1). Coupling of the linker was carried out by the treatment of **1** with Boc-NH-*p*ADA-OH (2 equiv.), a mixture of HBTU/Oxyma Pure (3.9 and 4 equiv., respectively) and DIPEA (8 equiv.). The beads were shaken for 2 h at rt, then they were washed thrice with DMF. Subsequently, the peptide **2a** was cleaved from the resin using a cleavage cocktail of 1,1,1,3,3,3-hexafluoro-2-propanol/dichloromethane (HFIP/DCM) (2 mL, *v*:*v* = 20:80). The beads were mixed for 1 h at rt and washed twice with the cleavage cocktail, then the liquid phase was collected in a round-bottomed flask. The solvent was removed using a rotary evaporator. The resulting peptide was precipitated using cold diethyl ether and collected by centrifugation. After cleavage, the coupling of 4-amino-2,6-dimethylheptane (2.5 equiv.) on the C-terminus was performed using benzotriazole-1-yl-oxy-tris-pyrrolidino-phosphonium hexafluorophosphate (PyBOP) (2.5 equiv.) and DIPEA (5 equiv.) in DMF. The reaction mixture was stirred for 1 h at rt, the solvent was removed under a vacuum and the product was collected by precipitation in cold diethyl ether. Global deprotection of the peptide was performed by treatment of the peptide with trifluoroacetic acid/water/triisopropyl silane (TFA/H_2_O/TIS) (2 mL, *v*:*v*:*v* = 95:2.5:2.5) for 1 h at rt. Later, the TFA was removed using a gentle air stream, and the resulting crude product **3a** was washed with cold diethyl ether and collected by centrifugation. The **3a** was purified with a Sep-Pak C18 35 cc Vac cartridge (10 g) (Waters, Etten-Leur, The Netherlands). The column was pre-conditioned with methanol (100 mL) and H_2_O (200 mL). The peptide was subsequently loaded onto the column and washed with water (200 mL) until the pH of the eluate became neutral. Then, **3a** was eluted using a mixture of H_2_O/ACN (*v*:*v* = 1:1, 4 × 20 mL), followed by two fractions of 20 mL ACN. The fractions containing the product were combined and lyophilized for further experiments. The final product **4a** was prepared by adding *trans*-cyclooctene-*N*-hydroxysuccinimide ester (TCO-NHS ester, 3 equiv.), triethylamine (10 equiv.) and H_2_O/ACN (2 mL, *v*:*v* = 1:1). The reaction was stirred for 3 h at rt. The crude compound was purified by the semi-preparative HPLC to provide **4a** as a white solid (8.6 mg, 2.0% yield). Analytical HPLC retention time of **4a**: *t*_R_ = 18.5 min. Purity > 95%. ESI-MS: *m*/*z*, calculated: 1340.73, found: 1341.00 [M + H]^+^.

#### 2.2.3. TCO-PEG_4_-fQWAVGH-NHCH[CH_2_CH(CH_3_)_2_]_2_ (**4b**)

**Compound 4b** was synthesized according to the protocol previously described for **4a**, with Boc-NH-PEG_3_-COOH (PEG_4_) as a linker (Scheme 1). The crude product was purified by semi-preparative HPLC to yield **4b** as a white solid (11.7 mg, 2.7% yield). Analytical HPLC retention time of **4b**: *t*_R_ = 18.4 min. Purity > 95%. ESI-MS: *m*/*z*, calculated: 1367.79 [M], found: 1368.79 [M + H]^+^ and 707.46 [M + 2Na]^2+^.

#### 2.2.4. DOTA-L(TCO)-pADA-fQWAVGH-NHCH[CH_2_CH(CH_3_)_2_]_2_ (**9a**)

**Scheme 2:**
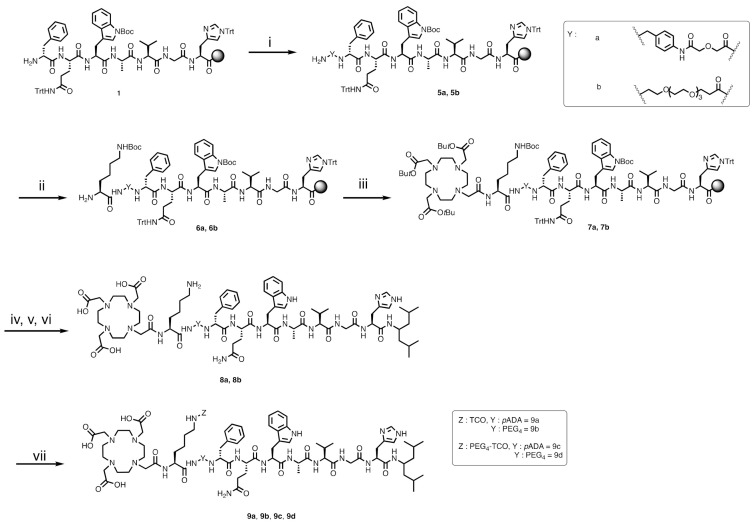
Synthesis of **9a**, **9b**, **9c** and **9d**. Reagents and conditions: (**i**) linker a or b, HBTU/Oxyma Pure, DIPEA, 2 h, rt, and 20% piperidine in DMF; (**ii**) Fmoc-L-Lys(Boc)-OH, HBTU/Oxyma Pure, DIPEA, 1 h, rt, and 20% piperidine in DMF; (**iii**) DOTA-tris(*t*Bu) ester, PyBOP, DIPEA, 2 h, rt; (**iv**) HFIP/DCM, 1 h, rt; (**v**) PyBOP, DIPEA, 4-amino-2, 6-dimethylheptane, DMF, 1 h; rt; (**vi**) TFA/H_2_O/TIS, 1 h, rt; (**vii**) TCO-NHS ester or TCO-PEG_4_ NHS ester, Et_3_N, H_2_O/ACN, 1 h, rt.

The first steps of the synthesis were performed from intermediate **1**, as described in the protocol above. The linker was protected with a Fmoc group instead of a Boc protecting group on the N-terminal position. Deprotection of Fmoc was carried out using 20% piperidine in DMF. The coupling of Fmoc-L-Lys(Boc)-OH was achieved with 4 equivalents of amino acid, HBTU/Oxyma Pure (3.9 and 4 equiv., respectively) and DIPEA (8 equiv.). The beads were shaken for 1 h at rt. After coupling, the beads were washed with DMF (3 × 1 mL), and Fmoc deprotection was performed as reported above. The coupling of 1,4,7,10-tetraazacyclododecane-1,4,7,10-tetraacetic acid tris tert-butyl ester (DOTA-tris(*t*Bu) ester, 3 equiv.) was realized in the presence of PyBOP (3 equiv.), DIPEA (6 equiv.) and DMF (3 mL) (Scheme 2). The reagents and the resin were mixed for 2 h at rt. The beads were washed with DMF (3 × 1 mL), followed by the cleavage of the peptide from the solid support, the coupling of 4-amino-2, 6-dimethylheptane on histidine, global deprotection, C18 Sep-Pak purification and conjugation of the TCO-NHS ester, as described above. Compound **9a** was purified by semi-preparative HPLC to give a white solid (28.2 mg, 1.2% yield). Analytical HPLC retention time of **9a**: *t*_R_ = 14.5 min. Purity > 98%. ESI-MS: *m*/*z*, calculated: 1855.00 [M], found: 928.51 [M + 2H]^2+^.

#### 2.2.5. DOTA-L(TCO)-PEG_4_-fQWAVGH-NHCH[CH_2_CH(CH_3_)_2_]_2_ (**9b**)

After the conjugation of TCO, the crude product was purified by semi-preparative HPLC to obtain **9b** as a white solid (14.7 mg, 0.6% yield). Analytical HPLC retention time of **9b**: *t*_R_ = 15.5 min. Purity > 95%. ESI-MS: *m*/*z*, calculated: 1882.06 [M], found: 1883.13 [M + H]^+^, 941.88 [M + 2H]^2+^, 952.87 [M + Na + H]^2+^ and 963.93 [M + 2Na]^2+^.

#### 2.2.6. DOTA-L(PEG_4_-TCO)-pADA-fQWAVGH-NHCH[CH_2_CH(CH_3_)_2_]_2_ (**9c**)

TCO-PEG_4_ NHS ester was coupled to **8a** to give **9c**, following the same protocol described above (Scheme 2). Compound **9c** was purified by semi-preparative HPLC and was obtained as a white solid (17.2 mg, 0.7% yield). Analytical HPLC retention time of **9c**: *t*_R_ = 15.6 min. Purity > 94%. ESI-MS: *m*/*z*, calculated: 2102.15 [M], found: 1051.91 [M + 2H]^2+^.

#### 2.2.7. DOTA-L(PEG_4_-TCO)-PEG_4_-fQWAVGH-NHCH[CH_2_CH(CH_3_)_2_]_2_ (**9d**)

The TCO-PEG_4_ NHS ester was coupled to **8b** and purification by semi-preparative HPLC provided **9d** as a white solid (18.6 mg, 0.7% yield) (Scheme 2). Analytical HPLC retention time of **9d**: *t*_R_ = 14.7 min. Purity > 97%. ESI-MS: *m*/*z*, calculated: 2129.20 [M], found: 1065.63 [M + 2H]^2+^.

#### 2.2.8. Tetrazine-Sulfo Cyanine 5 (Tz-sCy5)

The synthesis of Tz-Cy5 was performed, following the protocol described by Sasmal and coworkers [22]. The characteristics of the compound are provided in the Supplementary Materials.

#### 2.2.9. Synthesis of **10**

Compound **10** was prepared by reacting **4a** (2.01 mg, 1.49 µmol) and **Tz-sCy5** (1.33 mg, 1.1 equiv.). Both starting materials were dissolved in H_2_O/ACN (*v*:*v* = 1:1) and incubated at 37 °C for 10 min in an Eppendorf tube protected from light. The reaction mixture was purified by the semi-preparative HPLC to give **10** as a blue solid (2 mg, 63% Yield). Analytical HPLC retention time: *t*_R_ = 16.0 min. Purity > 92%. ESI-MS: *m*/*z*, calculated: 2124.00 [M], found: 1062.63 [M + H]^+^.

#### 2.2.10. Synthesis of **11**

Compound **11** was synthesized by following the experimental method reported above for **10**. Reaction of **4b** (2.5 mg, 1.80 µmol) and **Tz-sCy5** (1.6 mg, 1.1 equiv.) provided **11**, after purification and lyophilization, as a blue solid (1.8 mg, 46% yield). Analytical HPLC retention time: *t*_R_ = 15.9 min. Purity > 91%. ESI-MS: *m*/*z*, calculated: 2151.06 [M], found: 2153.88 [M + H]^+^ and 1077.04 [M + 2H]^2+^.

#### 2.2.11. Synthesis of **12**

Compound **12** was synthesized by following the experimental method reported above for **10**. Reaction of **9c** (4.3 mg, 2.04 µmol) and **Tz-sCy5** (1.8 mg, 1.1 equiv.) provided **12**, after purification and lyophilization, as a blue solid (1.33 mg, 23% yield). Analytical HPLC retention time: *t*_R_ = 15.2 min. Purity > 90%. ESI-MS: *m*/*z*, calculated: 2885.42 [M], found: 1442.92 [M + 2H]^2+^ and 969.63 [M + Na + H]^2+^.

#### 2.2.12. Synthesis of **13**

Compound **13** was synthesized by following the experimental method reported above for **10**. Reaction of **9d** (3 mg, 1.41 µmol) and **Tz-sCy5** (1.26 mg, 1.1 equiv.) provided **13**, after purification and lyophilization, as a blue solid (1.21 mg, 29% yield). Analytical HPLC retention time: *t*_R_ = 14.7 min. Purity > 96%. ESI-MS: *m*/*z*, calculated: 2912.48 [M], found: 1456.22 [M + 2H]^2+^ and 971.21 [M + 3H]^3+^.

#### 2.2.13. Synthesis of **14**

Compound **14** was synthesized by following the experimental method reported above for **10**. The reaction of **9a** (3 mg, 1.60 µmol) and **Tz-sCy5** (1.44 mg, 1.1 equiv.) provided **14**, after purification and lyophilization, as a blue solid (3.35 mg, 79% yield). Analytical HPLC retention time: *t*_R_ = 13.9 min. Purity > 95%. ESI-MS: *m*/*z*, calculated: 2638.28 [M], found: 1319.49 [M + 2H]^2+^ and 880.08 [M + 3H]^3+^.

#### 2.2.14. Synthesis of **15**

Compound **15** was synthesized by following the experimental method reported above for **10**. Reaction of **9b** (2.88 mg, 1.53 µmol) and **Tz-sCy5 (**1.37 mg, 1.1 equiv.) provided **15**, after purification and lyophilization, as a blue solid (1.89 mg, 46% yield). Analytical HPLC retention time: *t*_R_ = 14.7 min. Purity > 97%. ESI-MS: *m*/*z*, calculated: 2665.34 [M], found: 1332.81 [M + 2H]^2+^ and 888.54 [M + 3H]^3+^.

### 2.3. Radiolabeling of ***12***, ***13***, ***14*** and ***15*** with ^111^In

All ^111^In-labeled conjugates were prepared by adding 100 MBq of ^111^InCl_3_ (62.2 μL) to a solution of 1 nmol of peptide (**12**, **13**, **14** or **15**), ascorbic acid/gentisic acid (Gz/Asc) (10 μL, 50 mM), sodium acetate (1 μL, 2.5 M, pH 8) and H_2_O (60.8 μL). The final mixture, with a pH of 4.5, was incubated for 20 min at 90 °C. The reaction was monitored by instant thin-layer chromatography (iTLC) on silica gel impregnated glass-fiber sheets and with a solution of sodium citrate (0.1 M, pH 5) as an eluent. The reaction mixture was left to cool down for 5 min and diethylenetriaminepentaacetic acid (DTPA) (5 μL) was added to complex free ^111^In. Then, the radiochemical yield of the ^111^In-labeled peptides was determined using radio-HPLC.

### 2.4. Determination of the Distribution Coefficients (LogD_7.4_)

Distribution coefficients (LogD_7.4_) for the ^111^In-labeled compounds were determined by a shake-flask method. The experiments were performed in triplicate for each radioligand. A sample containing the radioligand (0.5–2.0 MBq) was dissolved in 1 mL solution of phosphate-buffered saline (0.01 M, pH 7.4) and *n*-octanol (*v*:*v* = 1:1). The vials were vortexed vigorously and then centrifuged at 10,000 rpm for 3 min, for phase separation. Samples (10 μL) of each phase were taken out and analyzed using a gamma counter. LogD_7.4_ values were calculated, using the following equation: LogD_7.4_ = log {(counts in *n*-octanol phase)/(counts in PBS phase)}.

### 2.5. Stability Studies in PBS and Mouse Serum

Stability in PBS was determined by incubating 20 µL of the labeled compounds (∼11 MBq) in PBS (80 µL) at 37 °C. Radiochemical purity was determined by iTLC at 30 min, 1 h, 2 h and 4 h. Stability in the serum was carried out by adding 70 µL of the labeled compound (∼35 MBq) to 330 µL of mouse serum (Merck, Haarlerbergweg, The Netherlands). The mixture was incubated at 37 °C. At different time points (30 min, 1 h, 2 h and 4 h), an aliquot of the mixture (50 µL) was added to 50 µL ACN. The vial was vortexed and centrifuged at 10,000 rpm for 20 min and the supernatant was analyzed by iTLC.

### 2.6. Cell Culture and Competition Binding Assay

GRPR-positive human-derived prostate adenocarcinoma epithelial PC-3 cells (ATCC, Manassas, VA, USA) were cultured in Ham’s F-12K (Kaighn’s) Medium (Gibco, Paisley, UK) supplemented with 10% fetal bovine serum, penicillin (100 units/mL) and streptomycin (100 µg/mL). Cells were routinely passaged and grown in tissue culture flasks at 37 °C in a humidified atmosphere with 5% CO_2_.

The affinity of all six probes for GRPR was determined using a competition binding assay. PC-3 cells were seeded in 12-well plates (2.5 × 10^5^ cells/well) one day prior to the experiment. The next day, cells were washed with warm Dulbecco’s phosphate-buffered saline (PBS) (Gibco). Subsequently, cells were incubated for 1 h at 37 °C with 0.5 mL incubation medium (Ham’s F-12K, 20 mM HEPES, 1% BSA, pH 7.4) containing 10^−9^ M [^111^In]In-NeoB, in the presence or absence of increasing concentrations (10^−12^ to 10^−6^ M) of one of the six unlabeled probes or NeoB (positive control). After incubation, the medium was removed and cells were washed twice with cold PBS. To determine the amount of activity that was taken up by the cells, cells were lysed using 1 M NaOH for > 20 min at rt, then collected and measured in a γ-counter. To determine the amount of radioactivity added per well, samples of the incubation medium containing 10^−9^ M [^111^In]In-NeoB were also measured. The results are expressed as the percentage of added dose (%AD) and were normalized to the uptake of [^111^In]In-NeoB (in the absence of cold compound). Data represent the mean ± standard deviation (SD) of triplicate wells.

### 2.7. Animal Model

Seven-week-old male Balb/c nu/nu-specific and opportunistic pathogen-free (SOPF) mice (Janvier Labs, Le Genest-Saint-Isle, France) were housed in individually ventilated cages, with 4 mice per cage. Upon arrival, mice were acclimated for 1 week, with access to food and water ad libitum. Mice were subcutaneously inoculated on the right shoulder with PC-3 cells (5 × 10^6^ cells suspended in 100 µL of 1/3 Matrigel (Corning Inc., Corning, NY, USA*)* and 2/3 Hank’s balanced salt solution (Gibco). PC-3 xenografts were allowed to grow for 3 weeks. Tumor sizes were 391 ± 173 mm^3^ at the start of the studies. All animal experiments were approved by the Animal Welfare Committee of the Erasmus MC and were conducted in agreement with institutional guidelines (license number: AVD101002017867, 28 September 2017).

### 2.8. In Vivo SPECT/CT Imaging Studies

Mice (*n* = 3 per probe) were intravenously injected in the tail vein with 200 µL of Kolliphor^®^ HS 15 (Merck, Haarlerbergweg, The Netherlands) in PBS (0.06 mg/mL) containing [^111^In]In-**12** (13.40 ± 0.76 MBq, ~670 pmol) or [^111^In]In-**15** (17.50 ± 1.78 MBq, ~875 pmol). To determine the receptor specificity of the probes, one additional animal was injected with [^111^In]In-**12** (9.78 MBq, 489 pmol) or [^111^In]In-**15** (10.17 MBq, 509 pmol), plus an excess of unlabeled NeoB (150 nmol). Two hours post-injection (p.i.), mice were imaged in a prone position on a heated bed under 2% isoflurane/O_2_ anesthesia, in a dedicated small-animal PET/SPECT/CT scanner (VECTor^5^CT scanner, MILabs B.V., Utrecht, The Netherlands) with a high sensitivity pinhole collimator (XXUHS-M, 3.0 mm pinhole diameter). Whole-body SPECT images (transaxial field of view (FOV) 54 mm) were acquired over 30 min using a spiral scan in normal scan mode, in list-mode acquisition. This was followed by a whole-body CT scan within 5 min, with the following imaging settings: full angle scan, angle step 0.75 degrees, normal scan mode, 50 kV tube voltage, 0.21 mA tube current, 500 µm aluminum filter. Reconstruction of the SPECT images was performed using the similarity-regulated OSEM method and MLEM method (MILabs Rec 11.00 software, MILabs B.V., Houten, The Netherlands) performing 9 and 128 iterations, respectively, at 0.8 mm^3^ resolution, using 173 keV ± 10% and 247 keV ± 10% energy windows for indium-111. Two adjacent background windows per photo peak were used for triple-energy window scatter and crosstalk correction. Reconstructed volumes of SPECT scans were post-filtered with an isotropic 3-dimensional Gaussian filter of 1 mm full width, at half-maximum. The CT and registered, attenuation-corrected SPECT images were analyzed using PMOD (PMOD 3.9, Zurich, Switzerland) and quantification was performed by placing volumes of interest (VOIs) around the tumors and kidneys. An Eppendorf tube filled with a solution of indium-111 of a known activity was measured to determine the calibration factor. The total activity measured in the VOI was divided by the volume of all VOI pixel values and multiplied by the calibration factor to obtain the percentage of injected activity per volume unit (%IA/mL).

### 2.9. Ex Vivo Biodistribution Studies and Optical Imaging

To determine the biodistribution of the compounds after imaging (~3 h p.i.), blood was collected via cardiac puncture under isoflurane/O_2_ anesthesia, after which the mice were sacrificed. The tumor and organs of interest (prostate, pancreas, spleen, liver, GI tract (stomach, small intestine, cecum, large intestine), kidneys, lungs, heart, muscle, bone, and brain) were excised, washed in PBS and blotted dry. The stomach, intestines and cecum were emptied of their contents. The tumor was cut in half; one half was fresh-frozen for further analysis and the other half was collected for ex vivo optical imaging and radioactivity measurements. After imaging, the blood, tumor, and relevant organs were weighed and measured in a γ-counter. To determine the total injected radioactivity per animal, samples of the injected solutions were measured as well. The percentage of injected dose per gram (%ID/g) was determined for each tissue sample and corrected for both the injected volume and %ID present at the injection site (the tail). Because the low weight of the prostate limited accurate organ weight measurements, the average prostate weight of all animals was used.

Before gamma counter measurements, the tumor half, pancreas, kidneys, lungs, small intestine, large intestine, liver, muscle, and bone were placed in two petri dishes and ex vivo optical imaging was performed with the IVIS Spectrum system (Perkin Elmer, Waltham, MA, USA) using the following settings for all measurements: FOV 12.6 cm, medium binning, f2, 0.5 s exposure with an excitation/emission filter of 640 nm/680 nm. Living Image version 4.5.2 software (Perkin Elmer) was used to perform data analysis by drawing a region of interest around the organ/tissue to quantify the radiant efficiency {(photons/second/cm^2^/steradian)/(μW/cm^2^)}.

### 2.10. Statistical Analysis

Statistical analysis was performed using GraphPad Prism version 5.01 (GraphPad Software Inc., San Diego, CA, USA). The half-maximal inhibitory concentration (IC_50_) values were estimated by the log (inhibitor) vs. the normalized response fitting routine. To compare the uptake values of the two probes studied in vivo, an unpaired *t*-test was used with the significance levels set at 5%. The results are represented as mean ± standard deviation (SD).

## 3. Results

### 3.1. Chemistry and Radiochemistry

Synthesis of the NeoB analogs was performed following solid-phase peptide synthesis (SPPS) protocols, using a standard Fmoc strategy. Coupling of the commercially available amino acids and linkers containing a Fmoc or a Boc protecting group was carried out with conventional coupling reagents, HBTU and Oxyma Pure (Scheme 1). Cleavage of the peptides from the solid support was achieved via treatment with a solution containing HFIP and DCM. Briefly, 4-Amino-2, 6-dimethylheptane was coupled on the C-terminal histidine by using PyBOP under basic conditions. Subsequent removal of the protecting groups from the compounds was achieved using a cocktail of TFA/H_2_O/TIS. Next, the peptides were purified by a C18 Sep-Pak cartridge, using H_2_O/ACN. Finally, compounds **4a** and **4b** were obtained in 2.0 and 2.7% yields, respectively, after coupling of the TCO-NHS ester under basic conditions and the purification of the products by semi-preparative HPLC.

Synthesis of the compounds **9a**, **9b**, **9c** and **9d** (Scheme 2) was achieved following a similar synthetic approach. Briefly, the Fmoc-protected linkers (*p*ADA and PEG_4_) were successfully coupled to the peptide sequence, followed by the removal of the Fmoc-protecting group with piperidine. The subsequent coupling of the lysine residue and DOTA chelator provided intermediates **7a** and **7b**. Cleavage of the peptides from the solid support, the coupling of 4-amino-2, 6-dimethylheptane on histidine, deprotection of the remaining protecting groups, and purification of the crude compounds by a C18 Sep-Pak cartridge afforded compounds **8a** and **8b**. The final peptides were obtained by coupling TCO-NHS ester or TCO-PEG_4_ NHS ester to the free amine on the side chain of the lysine residue. Compounds **9a**, **9b**, **9c** and **9d** were purified by semi-preparative HPLC and obtained in 1.2, 0.6, 0.7 and 0.7% yields, respectively.

**Figure 1:**
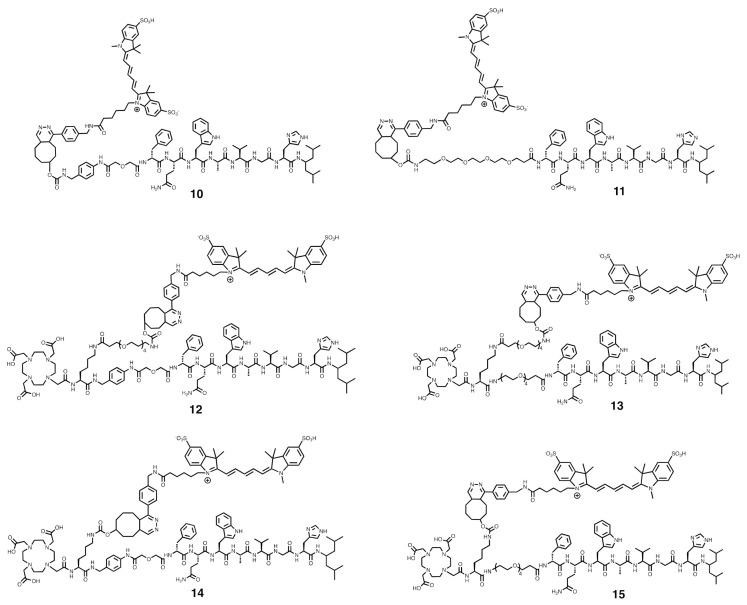
The chemical structures of the two mono-, **10** and **11**, and four dual-, **12**, **13**, **14** and **15** modality probes obtained via the IEDDA click reaction.

**Tz-sCy5** was obtained in 63% yield in a one-step synthesis under basic conditions [22]. With the six final NeoB analogs and **Tz-sCy5** in hand, we prepared two mono- and four dual-modality probes (Figure 1) via an IEDDA click reaction. The reaction was performed under mild conditions at 37 °C. The final compounds **10**, **11**, **12**, **13**, **14** and **15** were obtained in 63, 46, 23, 29, 79 and 46% yields, respectively, after semi-preparative HPLC purification.

Labeling of the final dual-modality probes was performed with ^111^InCl_3_ using a mixture of sodium acetate and Gz/Asc as scavengers to prevent radiolysis. The labeling efficiency was monitored by iTLC and radio HPLC (Table 1). The [^111^In]In-**12**, [^111^In]In-**13**, [^111^In]In-**14** and [^111^In]In-**15** were obtained in 97, 97, 92 and 96% yields, respectively.

All our probes exhibited a negative LogD_7.4_ value, proving their hydrophilicity. Stability studies in PBS showed that the percentage of intact labeled ligand considerably decreased over time, specifically for [^111^In]In-**14** (74.2% intact labeled ligand at 30 min, vs. 33.7% at 4 h) and [^111^In]In-**15** (83.1% intact labeled ligand at 30 min, vs. 57.1% at 4 h). However, all radiopeptides demonstrated high stability in mouse serum (> 94% intact labeled ligand at 4 h) (Table 1).

### 3.2. Binding Affinity Assays

[^111^In]In-NeoB was displaced from the GRPR binding site in PC-3 cells by the newly synthesized NeoB analogs. The results presented in Table 2 indicate that the binding affinity of the probes is 4.5 to 24.6-fold (21.9–118.7 nM) lower than the binding affinity of the parent peptide NeoB (4.8 nM) (Figure S1). The two dual-modality probes with the highest binding affinity (i.e., **12** and **15**) were selected for pilot in vivo pharmacokinetic characterization.

### 3.3. In Vivo SPECT/CT Imaging

**Figure 2:**
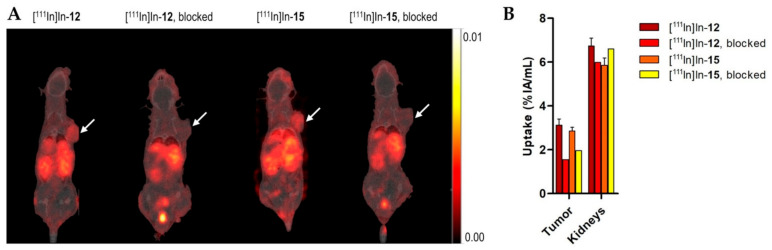
SPECT/CT imaging of [^111^In]In-**12** and [^111^In]In-**15** in PC-3-xenograft Balb/c nu/nu mice, 2 h p.i. (**A**) SPECT/CT images: overlay of a CT slice and the corresponding SPECT slice, on which the tumor cross-section is distinctly visible. The tumor is located on the right shoulder (white arrow). For the non-blocked groups, one representative image is shown. (**B**) Radioactivity uptake in tumor and kidney volumes of interest, determined from SPECT images, is expressed as a percentage of injected activity per mL (%IA/mL). The values for the unblocked groups are recorded as mean ± SD (*n* = 3).

SPECT/CT imaging of PC-3-xenografted mice 2 h after the administration of [^111^In]In-**12** or [^111^In]In-**15** demonstrated clear visualization of the tumor xenografts (Figure 2A). The uptake was GRPR-specific, since reduced tumor uptake was observed when the GRPR was blocked using an excess of unlabeled NeoB. As expected, uptake was observed in the kidneys and bladder as a result of the renal excretion of the probe and in the abdominal region, where GRPR-positive organs, such as the pancreas, are located. Quantification of the radioactivity in tissues of interest showed a similar uptake pattern for [^111^In]In-**12** and [^111^In]In-**15** in both the tumor and kidneys (Figure 2B), resulting in comparable tumor-to-kidney ratios (0.47 ± 0.05 for [^111^In]In-**12** and 0.49 ± 0.05 for [^111^In]In-**15**).

### 3.4. Biodistribution Studies

**Figure 3:**
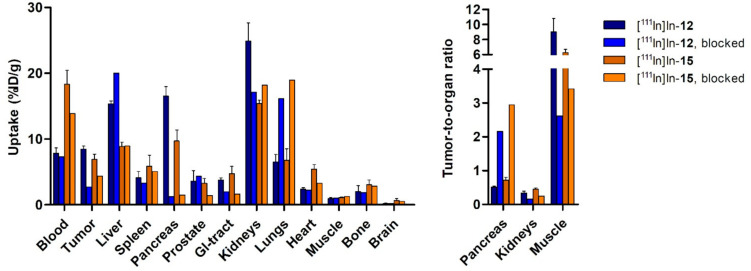
Ex vivo biodistribution (left) and tumor-to-organ ratios (right) of [^111^In]In-**12** and [^111^In]In-**15** in PC-3-xenograft Balb/c nu/nu mice after SPECT/CT scanning. Mice were injected intravenously with 1 nmol of [^111^In]In-**12** or [^111^In]In-**15,** alone or in combination with 150 nmol of non-labeled NeoB (blocked). Radioactivity uptake in tissues was calculated as a percentage of the injected dose per gram of tissue (%ID/g). Data for the unblocked group are presented as an average value ± SD (*n* = 3).

The results of the ex vivo biodistribution of [^111^In]In-**12** and [^111^In]In-**15** in PC-3-xenograft Balb/c nu/nu mice after SPECT/CT scanning are depicted in Figure 3 and Table S1. In agreement with the in vivo measurements, high radioactivity uptake was observed in the tumor with both probes (8.47 ± 0.46%ID/g and 6.90 ± 0.81%ID/g for probe [^111^In]In-**12** and [^111^In]In-**15**, respectively). In addition, the probes highly accumulated in the GRPR-expressing pancreas (16.55 ± 1.41%ID/g for [^111^In]In-**12**; 9.72 ± 1.63%ID/g for [^111^In]In-**15**). Both probes have similar tumor-to-muscle ratios, but the higher pancreas uptake of probe [^111^In]In-**12** resulted in a significantly lower tumor-to-pancreas ratio (*p* = 0.0131). In low-level GRPR-expressing organs, such as the GI tract and the prostate gland, moderate uptake was observed. Co-injection with an excess of unlabeled NeoB resulted in a strongly decreased uptake level of the probes in the GRPR-positive tumor and organs, indicating receptor specificity.

At the time of sacrifice, [^111^In]In-**12** and [^111^In]In-**15** were not completely cleared from the blood (7.83 ± 0.84%ID/g for [^111^In]In-**12**; 18.32 ± 2.12%ID/g for [^111^In]In-**15**). Probes were mainly eliminated via renal clearance; kidney uptake was 24.88 ± 2.78%ID/g for [^111^In]In-**12** and 15.38 ± 0.50%ID/g for [^111^In]In-**15**. The higher kidney uptake for probe [^111^In]In-**12** resulted in a lower tumor-to-kidney ratio for [^111^In]In-**12** (*p* = 0.0409). Furthermore, a relatively high radioactivity uptake was seen in the lungs (6.51 ± 1.15%ID/g for [^111^In]In-**12**; 6.77 ± 1.73%ID/g for [^111^In]In-**15**). This uptake was not GRPR-specific since a 2–3-fold higher uptake in this organ was also observed in animals that were co-injected with an excess of unlabeled NeoB (16.14%ID/g and 18.95%ID/g for [^111^In]In-**12** and [^111^In]In-**15**, respectively). Next to the lungs, unexpected high radioactivity levels were noticed in the liver (15.35 ± 0.43%ID/g for [^111^In]In-**12**; 8.87 ± 0.65%ID/g for [^111^In]In-**15**).

### 3.5. Ex Vivo Optical Imaging

**Figure 4:**
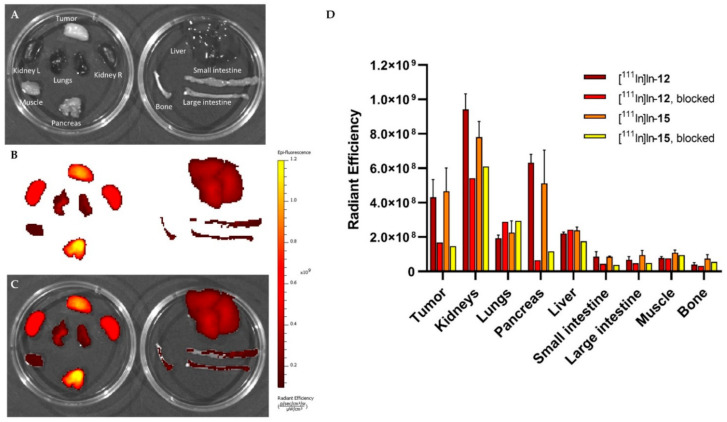
Ex vivo fluorescence imaging of a subset of organs. A representative example of (**A**) a photograph (organ tags in white), (**B**) a fluorescence image, and (**C**) their overlay, obtained with IVIS imaging of [^111^In]In-**15** in PC-3-xenografted Balb/c nu/nu mice after dissection. The scale bar shows the fluorescent signal intensities in radiant efficiency {(p/sec/cm^2^/sr)/(µW/cm^2^)}. (**D**) Quantified ex vivo radiant efficiencies of [^111^In]In-**12** and [^111^In]In-**15** uptake in tumor and organs (non-blocked (*n* = 3); blocked (*n* = 1)). Values for the unblocked group are presented as an average value ± SD.

A subset of organs and the tumor were also imaged by ex vivo fluorescence imaging, to confirm the localization of the dye (Figure 4). Here, the results showed that the fluorescence intensities of the organs generally followed the same trend as the radioactivity uptake. As can be deduced from Figure 4, high signal levels were measured in the tumor ([4.31 ± 1.03 and 4.66 ± 1.35] × 10^8^ p/sec/cm^2^/sr per µW/cm^2^ for [^111^In]In-**12** and [^111^In]In-**15** respectively), GRPR-positive pancreas ([6.32 ± 0.50 and 5.12 ± 1.93] × 10^8^ p/sec/cm^2^/sr per µW/cm^2^ for [^111^In]In-**12** and [^111^In]In-**15,** respectively) and kidneys ([9.41 ± 0.92 and 7.80 ± 0.92] × 10^8^ p/sec/cm^2^/sr per µW/cm^2^ for [^111^In]In-**12** and [^111^In]In-**15,** respectively) (Table S2). The intensities of bone and muscle are low, but muscle fluorescence is slightly higher for [^111^In]In-**15** than for [^111^In]In-**12** (*p* = 0.0456).

## 4. Discussion

Image-guided surgery of PCa can greatly aid surgeons to resect tumor tissues completely. Extensive research has been carried out on probes targeting the prostate-specific membrane antigen (PSMA), but it has been suggested that GRPR-targeted imaging probes may be of great value for PSMA-negative tumor lesions [23]. Moreover, the overexpression of GRPR typically occurs at an early stage of the disease, while PSMA is often associated with late-stage disease. Considering that image-guided surgery is particularly suitable for primary tumors, dual-modality probes targeting GRPR would be very attractive. NeoB exhibits a very high GRPR affinity and was introduced in the literature as a very promising peptide for GRPR-mediated radionuclide imaging and therapy [18,24]. Therefore, the chemical design of the dual-labeled probes is based on the amino acid sequence of the parent peptide NeoB. We herein described the synthesis of the new library of NeoB analogs with two linkers, the *p*ADA and PEG_4_ linkers. Those two linkers provide different physicochemical properties to the peptides, such as hydrophilicity, rigidity and spacing. In the chemical structure of compounds **4a** and **4b**, the original DOTA chelator was replaced by a TCO moiety, in order to preserve a chemical structure closer to that of the parent peptide. To add a TCO moiety to the original NeoB, a lysine residue was introduced between the peptide sequence and the DOTA chelator. This method has already been used by Li et al. and Zhang et al. for the insertion of a fluorescent dye into peptides [25,26]. Purification of the peptides by a C18 Sep-Pak cartridge before the coupling of TCO was implemented to remove the excess of TFA remaining from the removal of the protecting groups in the previous step. In fact, La-Venia and coworkers demonstrated that TCO is sensitive to acidic conditions and results in its change of conformation from TCO to CCO, the latter being less reactive toward Tz [27]. The final dual-labeled probes, containing a DOTA chelator and a fluorescent dye, were obtained via an IEDDA click reaction between the TCO coupled to the peptides and Tz coupled to the fluorescent dye, sCy5. The IEDDA reaction was chosen because of its fast kinetics, irreversibility, and stability of the generated product. The sCy5 dye was employed due to its strong fluorescence intensity and extinction coefficient, and excellent brightness [28,29]. Fluorescent dyes can show instability in aqueous solutions and a low pH. However, IEDDA provides the opportunity to synthesize the dual-labeled probes after obtaining the radiolabeled peptides containing the TCO moiety, preventing such instability from occurring. Another advantage of the click reaction is that it offers the possibility of synthesizing a large library of compounds in a single step.

The radiolabeling of the dual-labeled probes with ^111^InCl_3_ was successfully achieved with high RCYs. The negative LogD_7.4_ values were obtained for all the radiolabeled peptides. Compared to their radiolabeled NeoB analog, [^68^Ga]Ga-NeoB (LogD_7.4_ = −0.88 ± 0.02) [30], the introduction of the fluorescent dye to the peptides increased their hydrophilicity; therefore, negative LogD_7.4_ values were obtained for all the radiolabeled peptides. All radiolabeled compounds were stable in mouse serum, demonstrating their inertness toward peptidase digestion. However, the dual-labeled probes turned out to be less stable in PBS, demonstrating their sensitivity toward radiolysis [31].

The replacement of the DOTA chelator by a TCO moiety in compound **10** impaired the binding affinity of the peptide toward the receptor, probably due to a change in the conformation of the molecule. However, the introduction of a PEG_4_ linker instead of a *p*ADA linker did not substantially hamper the binding affinity of **11** toward GRPR, probably due to the flexibility offered by the linker. The introduction of two linkers in the four dual-labeled probes, **12**, **13**, **14** and **15,** induced changes in the conformation of the molecules, leading to a lower affinity of the compounds toward GRPR in comparison to the parent peptide, NeoB.

The results of the pilot in vivo study showed that probes [^111^In]In-**12** and [^111^In]In-**15** also possess good in vivo binding properties, depicted by a high tumor accumulation and a clear delineation of the xenograft on the SPECT scans. When compared to previous studies with NeoB [18,24], the fluorescent dye component of the probes noticeably influences the in vivo pharmacokinetic profile. The dual-modality probes have a prolonged blood circulation time, as high levels of radioactivity in the blood were observed at ~3 h post-injection. Hence, it could conceivably be hypothesized that the optimal tumor uptake and tumor-to-background levels may not have been reached in the current study. The observed uptake levels in the excretory organs (i.e., the liver and kidneys), compared to the biodistribution profile of the parent peptide NeoB, can also be attributed to the longer blood circulation time. Further work is needed to see if this can be improved, for example, by performing biodistribution and imaging studies at later time points.

The elevated radioactivity levels observed in the liver might be indicative of both renal and hepatobiliary routes of elimination. The elimination through hepatobiliary excretion was unexpected because this is unusual for probes with a non-lipophilic character [32]. However, TCO-Tz conjugates have a tendency to accumulate in hepatobiliary organs [33,34]. Furthermore, the involvement of this excretion pathway can possibly be attributed to aggregate formation due to increased “stickiness” (i.e., cohesive forces) of the dual-modality probes. This process could also be the cause of the unexpected uptake in the lungs, as the presence of aggregates in this organ can lead to capillary blockages, especially when high peptide amounts are injected, as was the case for the blocked animals [35]. Additional studies are needed that focus on mass optimization and practical and chemical implementations to reduce stickiness.

For a high image contrast on the preoperative scan and good visual inspection of the surgical field intraoperatively, it is crucial to have a high tumor-to-background ratio. As a rule of thumb, a ratio of 2 is reported in the literature [9]. In our study, the tumor-to-muscle ratios of the probes were found to be 9.04 ± 1.74 and 6.27 ± 0.44 for probe [^111^In]In-**12** and [^111^In]In-**15**, respectively. However, the tumor-to-blood ratio was not favorable. In addition, sufficient washout from the bladder (and thus, indirectly, the kidneys) is especially important in the image-guided surgery of PCa, because it is close to the surgical site [36]. A later imaging time point will most likely lead to reduced background signals in the blood and excretory organs.

To our knowledge, this is the second study, next to the investigations by Zhang et al. [26], about the in vivo evaluation of GRPR-targeted optical and nuclear dual-modality probes for PCa. The tumor uptake at ~2 h reported in our study (8.47 ± 0.46%ID/g and 6.90 ± 0.81%ID/g for probe [^111^In]In-**12** and [^111^In]In-**15**, respectively) was higher than the value that Zhang et al. obtained at ~1 h (5.50 ± 1.03%ID/g) for their GRPR-directed dual-modality probe, based on the RM2 backbone. However, they conducted a biodistribution study following a PET scan 1 h after injection. At this time point, their nuclear scan did benefit from a low background, due to a higher tumor-to-blood ratio compared to our designed probes, again arguing for optimization of the imaging time point in the future.

Further increasing the signal specificity is important for accurate intraoperative delineation of the target region and, more specifically, to distinguish between normal and tumor tissue [37]. Here, the specificity of our probes for the GRPR as a tumor target was demonstrated by a pilot blocking study using a single animal. Co-injection of an excess of unlabeled NeoB led to a decreased uptake in the tumor and the GRPR-expressing organs, such as the pancreas [38]. A reduction in activity levels in non-GRPR-expressing organs, such as the kidneys, on blocking might be due to a lower amount of activity injected in those animals. Despite the use of kolliphor as a surfactant in the solvent for the injections, this measure was not enough to compensate for the cohesive and adhesive properties of NeoB and the probes [18].

Another important finding was that the ex vivo fluorescence imaging confirmed the co-localization of the fluorescent and radioactive signal. This nicely illustrates the benefit of using a dual-modality probe with the same pharmacokinetics for 2 different purposes: pre- and intraoperative guidance. It is difficult to compare our measurements to previous studies with fluorescent agents targeting the GRPR because there is a potential for bias from the injected mass [39,40,41,42,43]. The injected amount can influence uptake, especially when receptor saturation levels are not yet reached. Nuclear imaging techniques have a slightly higher sensitivity than optical imaging. This means that in general, nmol amounts must be administered to allow fluorescence detection, while pmol amounts are often sufficient for nuclear detection. A further study with more focus on the optimal mass and specific labeling activity for both modalities is therefore suggested.

Since our study accommodated a pilot in vivo evaluation, future work should include a late-uptake analysis for all probes with a larger sample size for the blocked groups. Our study is supported by quantitative data obtained using various methods. Due to the image resolution and the fact that regions were drawn manually, the uptake values that were quantified by volume and count measurements from the SPECT/CT scans are less accurate than those obtained from the ex vivo biodistribution study. However, the ratios between organs calculated using both methods correlate well. Quantification of ex vivo optical data suffers from light attenuation. The differences between the organs relative to each other are therefore smaller overall, as is the difference between the two probes. The differences are therefore more evident from the radioactivity uptake levels measured in the ex vivo biodistribution study.

This study demonstrated the successful development and initial characterization of four promising dual-modality probes for the preoperative imaging and image-guided surgery of GRPR-positive PCa. Despite its exploratory nature, this study provides valuable insights into the influence of the incorporation of the sCy5 dye into the radiotracer NeoB on its binding affinity and pharmacokinetic properties. Although uptake was seen in the liver, lungs and pancreas, their location is not near the prostate and will therefore not interfere with the image-guided surgery. The prolonged blood circulation and high renal uptake require further evaluation of the optimal timing for imaging. Moreover, further in vivo preclinical evaluation with all four dual-modality probes will be performed to select the best probe for clinical translation. Overall, this study reinforces the idea that multimodal probes have very interesting properties to advance the field of image-guided surgery.

## Figures and Tables

**Table 1:** RCY, LogD_7.4_ and stability in PBS and mouse serum of [^111^In]In-**12**–**15**.

Compound	RCY (%)	Rf	Stability in PBS (%) ^a^ (*n* = 1)				Stability in Mouse Serum (%) ^a^ (*n* = 1)				Protein-Bound Fraction at 4 h (%)	Log D_7.4_ (*n* = 3)
			30 min	1 h	2 h	4 h	30 min	1 h	2 h	4 h		
[^111^In]In-**12**	97	0.04	85.8	80.1	75.0	73.1	96.3	96.4	96.5	96.5	7.0	−1.66 ± 0.01
[^111^In]In-**13**	97	0.06	87.4	87.5	86.2	84.4	98.1	97.9	97.6	97.3	5.6	−1.80 ± 0.01
[^111^In]In-**14**	92	0.07	74.2	48.5	30.9	33.7	94.6	94.1	94.0	94.1	7.1	−1.52 ± 0.1
[^111^In]In-**15**	96	0.06	83.0	71.8	67.0	57.1	96.9	96.7	96.9	97.1	6.5	−1.75 ± 0.09

^a^ Results are expressed as a percentage (%) of intact labeled ligand after incubation at 37 °C.

**Table 2:** IC_50_ values of the dual-modality probes, compared to the native NeoB peptide.

Compound	IC_50_ (nM)	Ratio Compound/NeoB
NeoB	4.8	
**10**	62.3	12.9
**11**	21.9	4.5
**12**	44.1	9.1
**13**	88.0	18.2
**14**	118.7	24.6
**15**	53.9	11.2

## Data Availability

Data can be requested by contacting the corresponding author.

## Supplementary Materials

The following supporting information can be downloaded at: https://www.mdpi.com/article/10.3390/pharmaceutics14010195/s1. Synthesis of **Tz-Cy5.,** Supplemental Figure S1: Inhibition of [^111^In]In-NeoB binding to PC-3 cells with probes **10, 11, 12, 13, 14, 15** and NeoB (as positive control), Table S1: Biodistribution of [^111^In]In-**12** and [^111^In]In-**15** in PC-3 xenograft Balb/c nu/nu mice after SPECT/CT scanning, Table S2: Fluorescent signal of [^111^In]In-**12** and [^111^In]In-**15** in organ/tissue samples after dissection.
