# Supplementary material for: Towards Complete Tumor Resection: Novel Dual-Modality Probes for Improved Image-Guided Surgery of GRPR-Expressing Prostate Cancer
Source: Pharmaceutics. 2022 Jan 14;14(1):195. doi: 10.3390/pharmaceutics14010195 (PMC8778164; doi:10.3390/pharmaceutics14010195)
Supplement: Supplementary file 1 [file pharmaceutics-14-00195-s001.zip › pharmaceutics-1510389-supplementary.pdf]

pharmaceutics

Supplementary Materials: Towards Complete Tumor Resection: Novel Dual-modality Probes for Improved Image-guided Surgery of GRPR-expressing Prostate Cancer

Maryana Handula, Marjolein Verhoeven, Kuo-Ting Chen, Joost Haeck, Marion de Jong, Simone U. Dalm and Yann Seimbille

Tetrazine-Sulfo Cyanine 5 (Tz-sCy5)

Tz-sCy5 was obtained as a blue solid (13.11 mg, 63% yield). Analytical HPLC retention time: t_R = 15.20 min. Purity > 94%. ESI-MS: *m/z*, calculated: 811.28 [M], found: 834.01 $[M+Na]^+$. ^1H NMR (400 MHz, D_2O): δ 10.00 (s, 1H), 7.84 (d, 2H, *J* = 7.7 Hz), 7.56-7.72 (m, 5H), 6.97-7.13 (m, 4H), 6.08 (t, 1H, *J* = 11.4 Hz), 5.83 (d, 1H, *J* = 13.2 Hz), 5.73 (d, 1H, *J* = 12.2 Hz), 4.20 (s, 2H), 3.77 (s, 1H), 3.63 (qt, 2H, *J* = 13.6, 6.7 Hz) 3.24 (s, 2H), 3.12 (q, 2H, *J* = 7.5 Hz), 2.17 (br, 1H), 1.52 (br, 2H), 1.29 (s, 3H) 1.27 (s,6H), 1.25 (s, 6H).

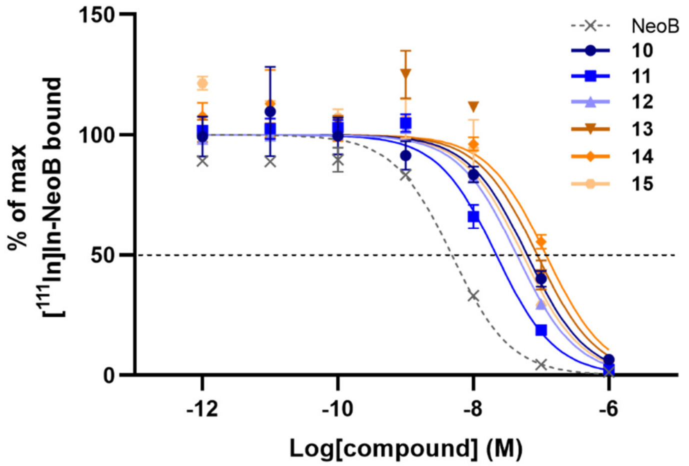

Figure S1. Inhibition of [^{111}In]In-NeoB binding to PC-3 cells with probes **10**, **11**, **12**, **13**, **14**, **15** and NeoB (as positive control). The dotted black line at 50% crosses the curves at the IC_{50} values. Data are presented as the average value of three wells.

Table S1. Biodistribution of [^{111}In]In-**12** and [^{111}In]In-**15** in PC-3 xenograft Balb/c nu/nu mice after SPECT/CT scanning. The uptake values are expressed as percentage injected dose per gram tissue (%ID/g).

**Organ/tissue**	**[^{111}In]In-12**		**[^{111}In]In-15**	
	**Non-blocked (*n* = 3)**	**Blocked (*n* = 1)**	**Non-blocked (*n* = 3)**	**Blocked (*n* = 1)**
Blood	7.83 ± 0.84	7.31	18.32 ± 2.12	13.87
Tumor	8.47 ± 0.46	2.72	6.90 ± 0.81	4.39
Pancreas	16.55 ± 1.41	1.26	9.72 ± 1.63	1.49
Prostate	3.56 ± 1.62	4.36	3.27 ± 0.74	1.42
Liver	15.35 ± 0.43	20.04	8.87 ± 0.65	8.93
Spleen	4.11 ± 0.94	3.32	5.86 ± 1.70	5.05
Stomach	3.22 ± 0.54	3.12	4.40 ± 1.04	3.48
Small intestine	4.40 ± 2.45	1.45	3.79 ± 0.69	1.97
Cecum	3.73 ± 0.40	2.35	5.21 ± 1.94	2.31
Large intestine	3.88 ± 0.40	2.06	5.60 ± 1.44	2.35
Kidneys	24.88 ± 2.78	17.11	15.38 ± 0.50	18.19
Lungs	6.51 ± 1.15	16.14	6.77 ± 1.73	18.95
Heart	2.39 ± 0.23	2.24	5.43 ± 0.69	3.27
Muscle	0.96 ± 0.16	1.04	1.10 ± 0.09	1.28
Bone	2.03 ± 0.89	1.87	3.05 ± 0.74	2.82
Brain	0.20 ± 0.03	0.20	0.67 ± 0.28	0.51
Tumor-to-organ ratios				
Tumor-to-blood	1.09 ± 0.15	0.37	0.38 ± 0.04	0.32
Tumor-to-pancreas	0.51 ± 0.03	2.16	0.72 ± 0.08	2.95
Tumor-to-prostate	2.86 ± 1.58	0.62	2.16 ± 0.46	3.09
Tumor-to-liver	0.55 ± 0.04	0.14	0.78 ± 0.04	0.49
Tumor-to-kidney	0.34 ± 0.05	0.16	0.45 ± 0.04	0.24
Tumor-to-muscle	9.04 ± 1.74	2.62	6.27 ± 0.44	3.42

Table S2. Fluorescent signal of [^{111}In]In-**12** and [^{111}In]In-**15** in organ/tissue samples after dissection. The signal is expressed as average radiant efficiency in 10^8 p/sec/cm^2/sr per μW/cm^2.

**Organ/tissue**	**[^{111}In]In-12**		**[^{111}In]In-15**	
	**Non-blocked (*n* = 3)**	**Blocked (*n* = 1)**	**Non-blocked (*n* = 3)**	**Blocked (*n* = 1)**
Tumor	4.31 ± 1.03	1.67	4.66 ± 1.35	1.47
Pancreas	6.32 ± 0.50	0.66	5.12 ± 1.93	1.17
Liver	2.20 ± 0.09	2.42	2.38 ± 0.21	1.75
Small intestine	0.85 ± 0.30	0.44	0.85 ± 0.04	0.38
Large intestine	0.68 ± 0.19	0.47	0.94 ± 0.28	0.49
Kidneys	9.41 ± 0.92	5.42	7.80 ± 0.92	6.09
Lungs	1.93 ± 0.18	2.88	2.25 ± 0.70	2.93
Muscle	0.78 ± 0.08	0.76	1.08 ± 0.16	0.96
Bone	0.40 ± 0.11	0.32	0.74 ± 0.24	0.55
Tumor-to-organ ratios				
Tumor-to-pancreas	0.69 ± 0.22	2.54	0.95 ± 0.18	1.25
Tumor-to-liver	1.96 ± 0.47	0.69	1.96 ± 0.57	0.84
Tumor-to-kidney	0.47 ± 0.16	0.31	0.59 ± 0.14	0.24
Tumor-to-muscle	5.69 ± 1.94	2.21	4.50 ± 1.87	1.54
